# Prospective evaluation of dose-escalated preoperative concurrent chemo-radiation with image guided-IMRT in locally advanced rectal cancers

**DOI:** 10.3332/ecancer.2023.1583

**Published:** 2023-07-26

**Authors:** Raunaq Puri, Madhup Rastogi, Ajeet Kumar Gandhi, Rohini Khurana, Rahat Hadi, Shantanu Sapru, Anshuman Pandey, Akash Agarwal, Anoop Kumar Srivastava, Surendra Prasad Mishra, Farhana Khatoon, Avinav Bharati, Vachaspati Kumar Mishra, Akanksha Manral, Prasoon Mishra

**Affiliations:** 1 Department of Radiation Oncology, Dr Ram Manohar Lohia Institute of Medical Sciences, Lucknow 226010, India; 2 Department of Surgical Gastroenterology, Dr Ram Manohar Lohia Institute of Medical Sciences, Lucknow 226010, India; 3 Department of Surgical Oncology, Dr Ram Manohar Lohia Institute of Medical Sciences, Lucknow 226010, India

**Keywords:** radiation dose escalation, treatment intensification, simultaneous integrated boost, neoadjuvant chemoradiation, locally advanced rectal cancers

## Abstract

**Purpose:**

To analyse the safety and efficacy of neoadjuvant chemoradiation (NACRT) with dose-escalated image-guided intensity modulated radiation therapy (IG-IMRT) in locally advanced (T3/4; T1-4N1-2) rectal cancers (LARCs).

**Materials and methods:**

Twenty patients with the diagnosis of LARC were recruited in this prospective interventional single-arm study treated by IG-IMRT with 45 Gray (Gy) in 25 fractions to elective nodal volumes and 55 Gy in 25 fractions to the gross primary and nodal disease with concurrent capecitabine 825 mg/m^2^ twice daily on radiotherapy days. Patients underwent total mesorectal excision 6–8 weeks post completion of NACRT followed by adjuvant chemotherapy (Capecitabine and oxaliplatin every 3 weekly for 6–8 cycles). Primary end point was acute toxicity assessment and secondary end points were pathological complete response (pCR) and loco-regional control (LRC).

**Results:**

Clinical T stage was T3:T4 in 19:1 and clinical N0:N1: N2 in 2:7:11 patients, respectively. With a median follow up of 21.2 months (13.8–25.6 months), 18 of 20 (90%) patients received the full course of treatment. Tumour and nodal downstaging was achieved in 78% and 84% of patients, respectively. pCR and overall complete response (defined as pCR and near CR) was achieved in 22.2% and 44.4% of patients, respectively. 2 (10%) patients completed NACRT, and achieved complete clinical response but refused surgery. Adjuvant chemotherapy course was completed by 17/18 (94.5%) patients. Grade 3 toxicities were observed in 2 (10%) patients during NACRT. All patients were disease-free at the time of the last follow up.

**Conclusion:**

Dose-escalation of NACRT therapy with IG-IMRT in LARC patients offers decent rates of pCR and overall response with excellent LRC and acceptable toxicities.

## Introduction

In today’s era, the treatment of locally advanced rectal cancer (LARC) is multimodal approach that includes surgery, chemotherapy and radiotherapy (RT). Neoadjuvant therapy typically involves the addition of radiation therapy in either the form of short-course or long-course regimens with each having their own advantages but neither being superior to the other [1–3], and its addition has shown to improve pathological complete response (pCR) rates, resectability, locoregional control (LRC) and potential improvement of sphincter preservation rates [4–6].

Recent advances have focused on treatment intensification with an attempt to further consolidate and improve outcomes. Treatment intensification may be done by many strategies including the addition of neoadjuvant chemotherapy to short-course or long-course chemotherapy, or the radiation dose escalation of the long-course neoadjuvant chemoradiotherapy [7–9].

However, short-course RT may lead to higher long-term complication rates [10] and long-course chemoradiation may be preferable particularly in patients with higher *T* stage and *N* stage as it leads to better downstaging of disease [1, 2].

Long-course chemoradiotherapy involves the dose prescription of 45–50.4 Gray (Gy) at 1.8 Gy per fraction with concurrent capecitabine on RT days. However, with this standard approach, the pCR rates achieved are 10%–20% in various randomised control trials and poor LRC rates are seen in patients with node- positive disease, particularly lateral pelvic wall lymph node disease and other nodal sites which are not routinely addressed surgically [4, 11]. pCR is defined as a complete response on pathological evaluation as ypT0N0M0, and it has been shown to be indicative of a prognostically favourable biological tumour profile and have better long-term outcomes including local recurrence, disease free and overall survival with less propensity for distant metastases [12, 13].

Radiation dose escalation can potentially radiate tumour volumes with relatively higher doses and treat microscopic spread to regional lymph nodes at higher doses than the current standard of care [14–16]. Multiple studies have explored the role of dose escalation and have been able to achieve higher pCR and local control rates although with the use of conventional techniques of radiation, they have been associated with higher acute and long-term toxicities [13, 17].

The use of image-guided intensity modulated radiation therapy (IG-IMRT) technique has enabled sparing of critical surrounding organs at risk (OARs) with the possibility of dose escalation and feasibility of prescribing different volumes to different doses and different doses per fractions [18, 19]. The use of this technique has been applied in this study to escalate radiation doses to volumes with high tumour burden while sparing the critical surrounding OARs.

However, the reported studies show significant variation in terms of radiation doses used and high variability in the pCR reports [20–22]. Several of the studies are also retrospective in nature and a study of this kind in a population of Indian ethnicities is lacking.

Our primary end point was to prospectively assess the acute toxicity rates and secondary end points included evaluation of the pCR rates and LRC rates with dose escalated IG-IMRT and radical surgery in our setup.

## Materials and methods

### Study design

The present study was a phase II single-arm, prospective, interventional, cohort study conducted between January 2021 and July 2022 in a tertiary cancer care institute in North India. The study was approved by the Institutional Ethics Committee (IEC No. 163/20) and written informed consent was taken from all patients prior to the enrolment.

### Clinical evaluation

All eligible patients underwent a complete detailed history and thorough clinical examination with digital rectal examination at the first visit. Baseline routine haematological, renal function tests, liver function tests, serum electrolytes, coagulation profile, viral markers, and serum carcinoembryonic antigen (CEA) was done. All patients then underwent a colonoscopy and biopsy with documentation including distance from anal verge, type of growth and extent of growth. Imaging studies at baseline included contrast-enhanced magnetic resonance imaging (MRI) pelvis, contrast-enhanced computed tomography (CT) abdomen or triple phase CT abdomen and either chest X-ray (P/A view) or contrast-enhanced CT thorax for routine chest imaging. All patients, at baseline were referred to either surgical gastroenterology or surgical oncology for future surgical planning.

### Radiotherapy

#### Simulation and planning

Patients were subjected to a simulation study with immobilisation in a supine position with hands above chest with the use of a knee rest as a positioning device. Contrast-enhanced CT simulation was performed on Siemens Somatom 16 slice CT simulator. Bladder protocol was followed which entailed intake of 500 mL water 30–45 minutes before simulation and patient being asked to hold urine until simulation was complete and subsequently before each treatment to limit inter-fraction or intra-fraction variability. A tiny radio-opaque marker was placed at the perineum to facilitate delineation. 70–100 mL of non-ionic contrast material was used, and the patient was scanned in a CT scanner, with 3 mm slice thickness. CT image data was transferred to the treatment planning station (MONACO SIM Version 5.11.03) via DICOM version.

Target volumes were contoured in accordance with Valentini *et al* [23] guidelines with minor modifications. Primary disease volumes contoured were gross tumour volumes (GTV) comprising of GTV primary which included gross disease of the rectum consistent with findings of clinical examination, proctosigmoidoscopy and radiological imaging. GTV nodal included involved nodes on radiological imaging with short axis diameter ³10 mm and with high-risk (HR) features (distorted architecture, indistinct nodal margins, irregular nodal capsular enhancement, and presence of necrotic/cystic areas). The clinical target volumes (CTV) were divided into the HR and low-risk (LR) volumes. HR CTV included GTV primary with a 2 cm cranio-caudal margin and a 1.5 cm axial margin and included the entire mesorectum, and GTV nodal with an isotropic margin of 10 mm. The LR CTV included the elective nodal volumes (CTV N_Elect_) which included the bilateral internal iliac, external iliac (if indicated), obturator, presacral and inguinal lymph node (if indicated). The contouring of elective nodal volumes included HR regional nodal regions which were grossly negative and was generated based on guidelines from Taylor *et al* [24].

The planning target volumes (PTV) included the HR PTV which included the HR CTV with a 1 cm isotropic margin and the LR PTV included the LR CTV with a 0.7 cm isotropic margin. The OAR were drawn as per the Radiation Therapy Oncology Group (RTOG) guidelines [25] for organ delineation in pelvic RT.

#### Dose prescription

RT was given over 5 weeks at 5 fractions per week in a total of 25 fractions by IMRT technique using the ELEKTA INFINITY linear accelerator.

While the LR PTV was given a dose of 45 Gy in 25 fractions at 1.8 Gy per fraction, the HR PTV was given a dose of 55 Gy in 25 fractions at 2.2 Gy per fraction, 5 fractions per week (Monday to Friday) over 5.5 weeks.

The plan objective was that at least 95% volume of PTV should be covered by the 95% isodose line. Position verification was done using kilo-voltage cone beam CT image verification and was done daily for the first 3 days and then twice per week, with additional imaging as per clinical discretion.

**Figure 1.**
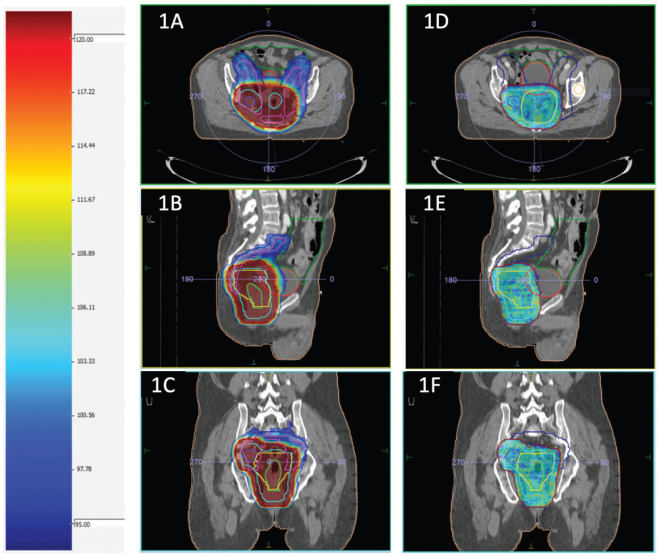
(a–c) and (d–f) shows dose colour wash of the LR and HR PTV, respectively.

Planning parameters for IMRT planning normal tissue planning goals for IMRT (dose constraints) were Bowel bag: V45 ≤ 65 cc; V40 < 100 cc; V35 < 180 cc; maximum dose (Dmax) < 55 Gy; V45 ≤ 195 cc (Soft constraint); Bladder: V45 < 25%; V40 < 40%; maximum dose (Dmax) < 55 Gy; Each femoral head: V40 < 40%; V45 < 25%; maximum dose (Dmax) < 50 Gy. The dose colour wash for HR and LR PTV for a representative case has been presented in Figure 1.

Acute toxicities were assessed according to Common Terminology Criteria of Adverse Event (CTCAE Version 5.0) from the day of start of the treatment up to 90 days every week during radiation therapy [26].

#### Concurrent chemoradiation (CTRT)

All patients received concurrent capecitabine at a dose of 825 mg/m^2^/day twice daily on days of radiation therapy (5 days a week) till the completion of radiation.

#### Surgery and pathological assessment

All patients were referred to either a gastrosurgeon or surgical oncologist and a staging re-evaluation was done with colonoscopy, contrast-enhanced MRI pelvis, contrast-enhanced CT abdomen and thorax. Surgery was performed within 6–8 weeks post completion of chemoradiation. Total mesorectal excision was performed in all patients with either a low anterior resection (LAR) or an abdominoperineal resection (APR) performed depending on the patient and tumour characteristics and surgeon’s choice.

Detailed histopathological examination and recording were done of the excised specimen including tumour size, histopathological subtype, grade and differentiation, margin status (proximal, distal and circumferential), number of nodes excised and those involved, presence of HR pathological features such as lympho-vascular invasion, perineural invasion or extra nodal extension and tumour downstaging. pCR rates were evaluated in accordance with the Modified Ryan Scheme for tumour regression score (based on the 2020 College of American Pathologist Protocol (Version 4.1.0.0) adopted by the AJCC 8th cancer staging manual) [27] Overall complete response (OCR) was defined in those patients who achieved a pCR or a near complete response (nCR).

#### Adjuvant chemotherapy

Adjuvant chemotherapy was given to all patients, irrespective of pathological response and 6–8 cycles of adjuvant chemotherapy [CAPOX (Capecitabine plus Oxaliplatin)] were given which included capecitabine 1,000 mg/m^2^ twice a day from day 1 to day 14, and Inj. Oxaliplatin 130 mg/m^2^ on day 1 and repeated every 21 days.

#### Post treatment follow up

Post treatment completion, visits were monthly for the first 3 months, and then 2 monthly for the next 6 months post-surgery. After this, patients were followed up every 3–4 months until 2 years after treatment, beyond which 6-monthly follow up was done with a minimum of 6 months follow-up attempted. The status of local disease and regional disease was clinically assessed at each follow up. Locoregional disease control assessment was done primarily by radiological and biochemical assessment which included contrast-enhanced MRI pelvis, contrast-enhanced CT abdomen and thorax, proctosigmoidoscopy, and serum CEA.

### Statistical analysis

Statistical analysis was done with Statistical Package for Social Sciences (SPSS v.25). Mean and SD were estimates of quantitative data. pCR was correlated by LRC rate. LRC was defined from the date of initiation of CTRT till the recurrence of local/regional disease.

## Results

Between January 2021 and July 2022, 20 patients of LARC were prospectively enrolled and all patients had histological diagnosis of adenocarcinoma. Patients aged 18–75 years with locally advanced stage IIA- IIIC disease (cT3-4N0 or any T N1-2 M0 as per AJCC 8th cancer staging system) with Karnofsky performance status 70 or above with normal routine haematological functions were included. Exclusion criteria included patients not fit for surgery or unwilling to undergo surgery or with involvement of external anal sphincter, presence of any distant metastasis, contraindication to capecitabine and its excipients, previous history of pelvic RT or previous chemotherapy, history of inflammatory bowel or rectum disease, pregnant woman, likely to be pregnant or nursing, any synchronous or metachronous malignancy or any active HIV, hepatitis B, hepatitis C positive patients or any uncontrolled co-morbidities (like DM, HTN, tuberculosis and coronary artery disease) likely to prevent the delivery of treatment.

Of 20 patients enrolled, 18 underwent definitive surgery and received full course adjuvant chemotherapy while 2 patients refused for surgery post completion of neoadjuvant chemoradiation (NACRT). They were assessed post completion of radiation treatment and their results have been given and discussed separately.

Thus, for the purpose of our study analysis, all 20 patients who received upfront NACRT were analysed for our primary endpoint i.e., acute toxicity assessment due to NACRT. Our secondary objectives included pCR rates for which definitive surgery was a mandatory inclusion. As the two patients deferred for definitive surgery, they have been excluded from the assessment for pCR although they have been included for assessment of LRC rates. The median follow up period was 21.2 months (range 13.8–25.6).

### Patient, tumour and treatment characteristics

Patient and tumour characteristics have been summarised in Table 1. Majority of the patients presented with chief complaint of bleeding per rectum in 18 (90%) patients and none of them presented with signs or symptoms of obstruction or obstructive disease. Majority patients (75%) presented with a low-lying tumour with the tumour arising from the lower rectum. The median duration of neoadjuvant chemo-radiotherapy was 36 days (Range: 34–37 days) with no significant treatment breaks or interruptions during radiation therapy. Planning parameters with dose-volume histogram analysis have been tabulated in Table 2.

### Toxicity analysis

The peak of acute radiation toxicities was noted in week 5, at the end of treatment. Toxicity profile have been listed in Table 3. Two patients (10%) developed grade 3 reactions during NACRT. One developed grade 3 radiation dermatitis possibly due to a very low-lying disease extending up to the anal verge, due to which the treatment volumes of the HR PTV extended up to the skin surface, while the other developed grade 3 diarrohea. Both grade 3 toxicities resolved spontaneously and on the first follow up post completion of radiation therapy with no persistence of symptoms. No patient developed any grade 4 toxicity. Overall patients tolerated radiation therapy well without any severe compliance issues or necessitating any treatment breaks. All 20 patients tolerated concurrent chemotherapy well and received full-dose chemotherapy without requiring any dose reductions or any blood transfusions/Granulocyte Colony Stimulating Factor (G-CSF) support during RT.

### Surgery characteristics

The median time to surgery from the end of radiation was 49 days (Range: 44–98 days). All surgeries were done by laparoscopic approach. Post definitive surgery, one patient developed post-surgical urinary obstruction and was managed by bilateral percutaneous nephrostomy while no patient developed any severe toxicity nor did any patient develop any anastomosis leakage post-surgery. The lower rates of surgical complication may be attributed to a higher number of patients with low-lying tumour.

### Pathological response and outcomes

Tumour and nodal down-staging were determined by comparing the pTNM stage to index cTNM stage. Overall pathological downstaging in primary tumour (T) Stage was seen in 14 patients (77%) while Nodal (N) downstaging was seen in 15 patients (83.3%). pCR was seen in 4/18 patients (22.2%), while the OCR was seen in 8/18 patients (44.4%). The pathological outcomes have been tabulated in Table 4.

### Adjuvant chemotherapy

As 2 patients did not undergo surgery, adjuvant chemotherapy was given in 18 patients with 17 patients (94.4%) completing entire course of adjuvant chemotherapy. Median number of cycles of chemotherapy was 6 (Range: 4–8). One patient who developed post-surgical urinary obstruction completed only received four cycles adjuvant chemotherapy. The median duration of adjuvant chemotherapy was 5.23 months, (Range: 3.3–6.3 months). 3 patient had delays during adjuvant chemotherapy (delay rate: 16%). No patient developed any significant grade 4 toxicity during adjuvant chemotherapy, although grade 1–3 toxicity were noted (48% patients developed grade 1–2 diarrhea and 16% had grade 3 diarrhea, 12% developed grade 1 or 2 hand-foot syndrome while 27% developed grade 1 or 2 neuropathy with none having grade 3 hand-foot syndrome or neuropathy) which resolved without causing any treatment breaks.

Two patients of 20 denied were definitive surgical intervention after thorough counselling of risk and prognosis agreed to continue routine follow up. Both these patients underwent colonoscopy and imaging for loco-regional and distant metastatic evaluation. As on last follow up, both patients had complete resolution of disease clinically and are healthy and asymptomatic.

No loco-regional relapses were found, and all patients are disease free at the time of the last follow up conferring a LRC of 100%.

## Discussion

The cohort in our study is representative of a locally advanced disease population with 90% of patients being stage III with a positive nodal disease at presentation. The current approach of intensified neoadjuvant chemoradiotherapy with escalated radiation dose followed by surgery and adjuvant chemotherapy is well tolerated and does not increase the incidence or severity of grade 3 or 4 toxicities as seen in our study population. Previous studies have reported rates of ≥ G3 toxicity 10%–30% with G2 toxicity ranging wide from 6.8% to 50% [9, 22, 28]. The low rates of > grade 3 toxicity are likely due to the use of IG-IMRT in our study.

The rationale of treatment intensification with dose escalation of radiation therapy is based on several dosimetric studies that showed elevating the dose to the gross disease would improve the tumour response. Although, such dose escalation may lead to increased dose to OARs and increased toxicity to the small bowel, bladder and femoral heads. With the use of IG-IMRT technique, using a simultaneous integrated boost (SIB) technique, we were able to irradiate two volumes to different overall doses and separate dose per fractions and thus able to increase the radiation dose to the primary and potentially spare the OARs to a major extent yielding improved oncological outcomes with lower toxicity rates.

Compared to the conventional regimen of 50.4 Gy at 1.8 Gy per fraction, the gross disease received a larger dose of 55 Gy and at a higher dose per fraction of 2.2 Gy per fraction representing an approximate 10% increase in the total radiation dose while the overall treatment time was shortened from the conventional 28 to 25 days as well. This amounted to a biologically equivalent dose (BED) of 67.10 Gy in comparison to the standard dose prescription of 50.4 Gy at 1.8 Gy per fraction receiving a BED of 59.47 Gy alone.

In a recently published systematic review of literature by Delishaj *et al* [28], dose escalation beyond a BED >70.7 Gy or EQD2 dose >58.9 Gy did not show any benefit in terms of improvement of pathological response or any survival benefit but was associated with higher toxicities, especially related to post-surgical complications and healing.

The elective treatment volume (LR volume) was given a dose of 45 Gy in 25 fractions at 1.8 Gy per fraction generally considered to be adequate for microscopic disease control, and in this manner, we were able to prescribe an optimal dose to a large volume which would be receiving a larger dose. The amount of bowel irradiated to higher doses was also reduced and with the help of IG-IMRT technique it led to the sparing of uninvolved small bowel and patients having improved wound and anastomotic healing rates and overall better post-surgical outcomes.

Compared to standard dose long-course radiation, the dose escalation of radiation led to an increased pCR rate with complete response in 22.2% patients, which is at the higher boundary of the reported pathological response rates in literature with conventional long-course NACRT. A retrospective study from our institute reported that a pCR of 10% was achieved in rectal cancer patients treated with standard dose-NACRT [29]. The OCR rates (including the pCR and nCR rates) achieved in the present study was 44.4% which has been correlated with disease-free and overall survival. The results of such similar trials are tabulated in Table 5.

While the total neoadjuvant therapy approach is gaining popularity with high pCR rates of nearly 28% reported by the RAPIDO and UNICANCER-PRODIGE 23 study groups, these have been at the cost of higher grade 3–4 serious adverse events of 38% in the RAPIDO trial and 27% in the PRODIGE 23 trial [7, 30]. Recently, the RAPIDO trialist groups reported their long-term results and the quality-of-life analysis and found that the total neo-adjuvant therapy group had a significantly higher nervous system (grade 3 as well as grade 1–2) and fatigue (grade 1–2) as compared to the standard chemoradiotherapy arm at 6 months of follow up. While these differences in the toxicities vanished at a longer follow up of 12 months, the experimental arm continued to have higher grade 1–2 nervous system toxicities [31].

One of the issues with the contemporary neo-adjuvant treatment is non-compliance of patients to surgery after good clinical and radiological response to induction treatment. In our analysis as well, two patients denied for surgery due to their own personal choice, but both agreed to continue routine follow-up with no further treatment and were declared as clinically complete responders. Both the patients presented with a lower primary tumour stage disease which could explain the response. Both patients did not develop any significant toxicities and did not have any treatment interruption, rather the reason for the unplanned non-compliance for surgery was due to the patients own unwillingness for a sphincter destructing surgery. This has been seen as a reason for non-compliance in various randomised trials and led to the genesis of the sphincter preserving and non-operative approaches for management of rectal cancers [30–33]. Our study approach may also need to be validated for wait-and-watch approach. Addition of neoadjuvant chemotherapy may further intensify the paradigm for an improved standard of care.

The strengths of this study focusses on the use of standardised contouring of CTV according to the recommended international consensus guidelines on CTV delineation contouring guidelines in rectal cancer given by Valentini *et al* [23] which ensures consistency and reproducibility of target volumes and allowed meaningful comparisons of results with other studies. Secondly, RT quality assurance was performed frequently for every patient within 1 week of patient starting treatment.

The main limitations of our study are the lack of randomisation and comparison of our novel IG-IMRT dose escalation technique with standard chemoradiation; and given the short follow up period, the need for a longer follow up data and larger sample size to estimate late toxicities and distant failures is imminent.

With all the information derived, we can say that radiation dose escalation of long-course NACRT to a dose of 55 Gy in patients of locally advanced rectal cancer using IG-IMRT is a safe and feasible option with good tolerability, minimal acute toxicities and excellent LRC and survival rates with our median follow up of 21.2 months.

## Conclusion

Dose-escalation of NACRT therapy with image-guided intensity modulated RT in locally advanced rectal cancer patients offers decent rates of pCR and overall response with excellent LRC and acceptable toxicities. The neo-adjuvant armamentarium for locally advanced rectal cancers have expanded in the last decade with incorporation of chemotherapy in the neo-adjuvant setting and the evolution of total neoadjuvant therapy, however, the best treatment option for a patient is arrived at by a multidisciplinary discussion. The current study approach of intensification with IG-IMRT needs further evaluation in well-designed phase III randomised controlled trials.

## Conflicts of interest statement

The authors have no conflicts of interest to declare.

## Funding declaration of all authors

None. We also did not receive any funding for the present study from any source.

## Author contributions

Conceptualization: RP, MR, AKG; Investigation and methodology: RP, MR, AKG, RK, RH, SS, SPM, AKS, AB, AP, AA; Supervision: MR, AKG, RK, SPM, AKS, AB; Data curation: RP, MR, AKG,RK, RH, SS, SPM, AKS, AB, AM, PM; Analysis and interpretation: MR, AKG, RK, RH, SS, SPM, AKS, AB,VKM, AM; Writing of the original manuscript: RP, MR, AKG; Writing of the review and editing: RP, MR, AKG, RK. All authors have proofread and approved the final version.

## Figures and Tables

**Table 1.** Patient and tumour characteristics.

Characteristics	Number (Percentage)
Age (years): median, range	40, 23–73
Gender (Male: females)	12 (60%): 8 (40%)
cT Stage (cT3: cT4)	19 (95%):1 (5%)
cN Stage (cN0: cN1: cN2)	2 (10%):7 (35%):11 (55%)
Stage group (cT3N0: cT3N1:cT3N2: cT4N1)	2 (10%): 6 (30%): 11 (55%): 1 (5%)
Overall stage (Stage II: Stage III)	2 (10%): 18 (90%)
Part of rectum involved – upper third: middle third: lower third	2 (10%): 3 (15%): 15 (75%)
Distance from Anal verge –Median, (Range)	3.75 cm, (0–8 cm)
Craniocaudal length – Median, (Range)	5.5 cm, (4–8 cm)
Preoperative CEA – Median, (Range)	1.73 (0.01–819) ng/mL
Grade– WD:MD:PD: mucinous	5 (25%): 6 (30%): 3 (15%): 6 (30%)
Extramural vascular invasion positive	4/20 (20%)
Mesorectal fat invasion	15/20 (75%)
Extra-mesorectal lymph node	8/20 (40%)
Baseline CRM status (Involved)	1/20 (5%)
Type of surgery – LAR: APR	4 (22%): 14 (78%)

Abbreviations- CEA: Carcinoembryonic antigen, WD: Well differentiated, MD: Moderately differentiated, PD: Poorly differentiated, CRM: Circumferential resection margin, LAR: Low anterior resection, APR: Abdominoperineal resection

**Table 2.** Dosimetric parameters of PTV and OAR’s.

		Dosimetric parameter		(Mean ± SD)
PTV_HR (55 Gy)		V_95%_		99.34 ± 0.63
PTV_LR (45 Gy)		V_95%_		99.27 ± 0.82
OAR	Dosimetric parameter		Dosimetric constraints	Accepted parameters (Mean ± SD)
Bladder	D_max_(Gy)		<55 Gy	58.86 ± 1.05
	V_50Gy_ (Gy)		<50% (Soft)	32.31 ± 8.44
Right femoral head	D_max_(Gy)		<50 Gy	46.58 ± 2.88
	V_40Gy_ (Gy)		<40%	3.53 ± 3.63
	V_45Gy_ (Gy)		<25%	0.56 ± 1.12
Left femoral head	D_max_(Gy)		<50 Gy	46.92 ± 2.98
	V_40Gy_ (Gy)		<40%	4.19 ± 3.72
	V_45Gy_ (Gy)		<25%	0.60 ± 1.3
Bowel bag	D_max_(Gy)		<55 Gy	53.68 ± 4.72
	V_45Gy_ (Gy)		<65 cc, <195 cc (Soft)	30.42 ± 13.46
	V_40Gy_ (Gy)		<100 cc	81.88 ± 28.13
	V_35Gy_ (Gy)		<180 cc	133.14 ± 46.17

**Table 3.** Toxicity assessment of NACRT (overall assessment of maximum toxicity).

		Grade 0	Grade 1	Grade 2	Grade 3	Grade 4	Overall (%)
Gastrointestinal	Perianal pain	12	7	1	0	-	8 (40)
	Diarrhea	8	7	4	1	-	12 (60)
	Proctitis	10	7	3	0	-	10 (50)
Genito-urinary	Cystitis	12	6	2	0	-	8 (40)
Skin	Radiation dermatitis	10	6	3	1	-	10 (50)
	Palmar-Plantar erythrodysesthesia	11	7	2	0	-	9 (45)
Hematological	Anaemia	11	7	2	0	-	9 (45)
	Neutropenia	12	6	2	0	-	8 (40)
	Thrombocytopenia	15	5	0	0	-	5 (25)

**Table 4.** Pathological outcomes and response.

Pathological outcomes and response	
Pathological outcomes and response	Result
Pathological T stage ypT0: ypTis: ypT1: ypT2: ypT3	4:2:2:6:4
Pathological N stage ypN0:ypN1:ypN2	16:1:1
Pathological response pCR: nCR: partial response: poor response	4 (22.2%): 4 (22.2%): 7 (38/8%): 3 (16.6%)
Lympho-vascular invasion: perineural invasion	5 (27.7%): 4 (22.2%)
Circumferential resection margin positive	1 (5.55%)

**Table 5.** Showing characteristics and outcomes from comparative studies.

Study	No. of patients	Prescription dose/No. of fractions		Median follow up (months)	pCR rates (%)	LRC rates (%)	Toxicity (%) (NACRT)		Surgical complication rates (%)
		Boost dose	Prophylactic dose				Gr2	>Gr3	
Zhu *et al* [35]	78	55/25	50/25	30	23.7	85.4 (at 3 years)	GI-14.1, Skin- 20.5	GI-10.3, Skin-17.9	17.1
Liu *et al* [36]	85	55/25	45–50/25	35.7	21.4	83.9 (at 3 years)	-	GI-5.2, GU-1.8	12.5
Yamashita *et al* [37]	60	55/25	45/25	44.7	17.0	90 (at 3 years)	GU-49	-	3.0
Chiloiro *et al* [38]	22	55/25	45/25	-	27.3	-	GU-22.7, GI-40	GI-22.7	-
Tey *et al* [39]	20	55/25	45/25	38.2	35.0	100 (at 2 years)	0	5	0
Zhao et al [40]	141	55/25	45/25	38.5	22.7	95.5 (estimated at 5 years)	-	GI-7.8	10.6
Present study	20	55/25	45/25	21.2	22.2	100	GI-40, GU-10, Skin-25	GI-5, Skin-5	5%

## References

[1] Ngan SY, Burmeister B, Fisher RJ (2012). Randomized trial of short-course radiotherapy versus long-course chemoradiation comparing rates of local recurrence in patients with T3 rectal cancer: Trans-Tasman radiation oncology group trial 01.04. J Clin Oncol.

[2] Bujko K, Nowacki MP, Nasierowska-Guttmejer A (2006). Long-term results of a randomized trial comparing preoperative short-course radiotherapy with preoperative conventionally fractionated chemoradiation for rectal cancer. Br J Surg.

[3] McLachlan SA, Fisher RJ, Zalcberg J (2016). The impact on health-related quality of life in the first 12 months: a randomised comparison of preoperative short-course radiation versus long-course chemoradiation for T3 rectal cancer (Trans-Tasman radiation oncology group trial 01.04). Eur J Cancer.

[4] Sauer R, Becker H, Hohenberger W (2004). Preoperative versus postoperative chemoradiotherapy for rectal cancer. N Engl J Med.

[5] Chen ET, Mohiuddin M, Brodovsky H (1994). Downstaging of advanced rectal cancer following combined preoperative chemotherapy and high dose radiation. Int J Radiat Oncol Biol Phys.

[6] Chao M, Gibbs P, Tjandra J (2005). Preoperative chemotherapy and radiotherapy for locally advanced rectal cancer. ANZ J Surg.

[7] Bahadoer RR, Dijkstra EA, van Etten B (2021). Short-course radiotherapy followed by chemotherapy before total mesorectal excision (TME) versus preoperative chemoradiotherapy, TME, and optional adjuvant chemotherapy in locally advanced rectal cancer (RAPIDO): a randomised, open-label, phase 3 trial. Lancet Oncol.

[8] Smith JJ, Chow OS, Gollub MJ (2015). Organ preservation in rectal adenocarcinoma: a phase II randomized controlled trial evaluating 3-year disease-free survival in patients with locally advanced rectal cancer treated with chemoradiation plus induction or consolidation chemotherapy, and total mesorectal excision or nonoperative management. BMC Cancer.

[9] Hearn N, Atwell D, Cahill K (2021). Neoadjuvant radiotherapy dose escalation in locally advanced rectal cancer: a systematic review and meta-analysis of modern treatment approaches and outcomes. Clin Oncol (R Coll Radiol).

[10] Erlandsson J, Fuentes S, Radu C (2021). Radiotherapy regimens for rectal cancer: long-term outcomes and health-related quality of life in the Stockholm III trial. BJS Open.

[11] Bosset JF, Collette L, Calais G (2006). Chemotherapy with preoperative radiotherapy in rectal cancer. N Engl J Med.

[12] Vecchio FM, Valentini V, Minsky BD (2005). The relationship of pathologic tumor regression grade (TRG) and outcomes after preoperative therapy in rectal cancer. Int J Radiat Oncol Biol Phys.

[13] Martin ST, Heneghan HM, Winter DC (2012). Systematic review and meta-analysis of outcomes following pathological complete response to neoadjuvant chemoradiotherapy for rectal cancer. Br J Surg.

[14] Gunther JR, Chadha AS, Shin US (2017). Preoperative radiation dose escalation for rectal cancer using a concomitant boost strategy improves tumor downstaging without increasing toxicity: a matched-pair analysis. Adv Radiat Oncol.

[15] Bosset JF, Calais G, Mineur L (2005). Enhanced tumorocidal effect of chemotherapy with preoperative radiotherapy for rectal cancer: preliminary results – EORTC 22921. J Clin Oncol.

[16] Appelt AL, Vogelius IR, Pløen J (2014). Long-term results of a randomized trial in locally advanced rectal cancer: no benefit from adding a brachytherapy boost. Int J Radiat Oncol Biol Phys.

[17] Arias F, Eito C, Asín G (2017). Fecal incontinence and radiation dose on anal sphincter in patients with locally advanced rectal cancer (LARC) treated with preoperative chemoradiotherapy: a retrospective, single-institutional study. Clin Transl Oncol.

[18] Bhide SA, Newbold KL, Harrington KJ (2012). Clinical evaluation of intensity-modulated radiotherapy for head and neck cancers. Br J Radiol.

[19] Pollard JM, Wen Z, Sadagopan R (2017). The future of image-guided radiotherapy will be MR guided. BJR.

[20] Teo MTW, McParland L, Appelt AL (2018). Phase 2 neoadjuvant treatment intensification trials in rectal cancer: a systematic review. Int J Radiat Oncol Biol Phys.

[21] Burbach JPM, Den Harder AM, Intven M (2014). Impact of radiotherapy boost on pathological complete response in patients with locally advanced rectal cancer: a systematic review and meta-analysis. Radiother Oncol.

[22] Zhao J, Hu W, Cai G (2016). Dosimetric comparisons of VMAT, IMRT and 3DCRT for locally advanced rectal cancer with simultaneous integrated boost. Oncotarget.

[23] Valentini V, Gambacorta MA, Barbaro B (2016). International consensus guidelines on clinical target volume delineation in rectal cancer. Radiother Oncol.

[24] Taylor A, Rockall AG, Reznek RH (2005). Mapping pelvic lymph nodes: guidelines for delineation in intensity-modulated radiotherapy. Int J Radiat Oncol Biol Phys.

[25] Gay HA, Barthold HJ, O’Meara E (2012). Pelvic normal tissue contouring guidelines for radiation therapy: a radiation therapy oncology group consensus panel atlas. Int J Radiat Oncol Biol Phys.

[26] Cancer Institute N Common terminology criteria for adverse events (CTCAE) common terminology criteria for adverse events (CTCAE); 2017. v5.0 [internet]. https://www.meddra.org/.

[27] Tang LH, Berlin J, Branton P (2016). Protocol for the examination of specimens from patients with primary carcinoma of the colon and rectum. http://www.cap.org.

[28] Delishaj D, Fumagalli IC, Ursino S (2021). Neoadjuvant radiotherapy dose escalation for locally advanced rectal cancers in the new era of radiotherapy: a review of literature. World J Clin Cases.

[29] Rath S, Gandhi AK, Rastogi M (2019). Long course neoadjuvant concurrent chemo-radiotherapy with or without pre-radiation induction chemotherapy in the management of rectal cancers: a mono-institutional retrospective analysis. Int J Radiat Oncol.

[30] Conroy T, Bosset JF, Etienne PL (2021). Neoadjuvant chemotherapy with FOLFIRINOX and preoperative chemoradiotherapy for patients with locally advanced rectal cancer (UNICANCER-PRODIGE 23): a multicentre, randomised, open-label, phase 3 trial. Lancet Oncol.

[31] Dijkstra EA, Hospers GAP, Kranenbarg EM (2022). Quality of life and late toxicity after short-course radiotherapy followed by chemotherapy or chemoradiotherapy for locally advanced rectal cancer – the RAPIDO trial. Radiother Oncol.

[32] Sun W, Al-Rajabi R, Perez RO (2020). Controversies in rectal cancer treatment and management. Am Soc Clin Oncol Educ Book.

[33] Peeters KCMJ, Velde CJH, Leer JWH (2005). Late side effects of short-course preoperative radiotherapy combined with total mesorectal excision for rectal cancer: increased bowel dysfunction in irradiated patients – a Dutch colorectal cancer group study. J Clin Oncol.

[34] Garcia-Aguilar J, Patil S, Gollub MJ (2022). Organ preservation in patients with rectal adenocarcinoma treated with total neoadjuvant therapy. J Clin Oncol.

[35] Zhu J, Liu F, Gu W (2014). Concomitant boost IMRT-based neoadjuvant chemoradiotherapy for clinical stage II/III rectal adenocarcinoma: results of a phase II study. Radiat Oncol.

[36] Liu X, Wang J, Hu K (2020). Neoadjuvant chemoradiotherapy or radiotherapy in patients aged 75 years or older with locally advanced rectal cancer. J Cancer.

[37] Yamashita H, Ishihara S, Nozawa H (2017). Comparison of volumetric-modulated arc therapy using simultaneous integrated boosts (SIB-VMAT) of 45 Gy/55 Gy in 25 fractions with conventional radiotherapy in preoperative chemoradiation for rectal cancers: a propensity score case-matched analysis. Radiat Oncol.

[38] Chiloiro G, Boldrini L, Meldolesi E (2019). MR-guided radiotherapy in rectal cancer: first clinical experience of an innovative technology. Clin Transl Radiat Oncol.

[39] Tey J, Leong CN, Cheong WK (2017). A phase II trial of preoperative concurrent chemotherapy and dose escalated intensity modulated radiotherapy (IMRT) for locally advanced rectal cancer. J Cancer.

[40] Zhao J, Liu X, Wang W (2019). Concomitant dose escalation with image-guided tomotherapy in locally advanced mid–low rectal cancer: a single-center study. Cancer Manag Res.

